# Longitudinal assessment of syringomyelia in Pomeranians

**DOI:** 10.3389/fvets.2024.1364464

**Published:** 2024-05-01

**Authors:** Koen M. Santifort, Ines Carrera, Paul J. J. Mandigers

**Affiliations:** ^1^IVC Evidensia Referral Hospital Arnhem, Neurology, Arnhem, Netherlands; ^2^IVC Evidensia Referral Hospital Hart van Brabant, Neurology, Waalwijk, Netherlands; ^3^Expertise Centre of Genetics, Department of Clinical Sciences, Faculty of Veterinary Medicine, Utrecht University, Utrecht, Netherlands; ^4^Vet Oracle Teleradiology, Norfolk, United Kingdom

**Keywords:** Chiari-like malformation, syringomyelia, syrinx, quantitative assessment, T1-weighted images, Pomeranian

## Abstract

**Introduction:**

Chiari-like malformation (CM) and syringomyelia (SM) are disorders that, in dogs, affect mainly small and toy breeds, including the Pomeranian. These disorders are linked to a great number of (owner-reported) clinical signs (ORCS) suggestive of pain. Aging was associated with an increased risk of having SM in several studies. However, there are only a few longitudinal studies that assess the presence and severity of CM/SM over time in CKCS dogs and progression of SM was linked to progression of clinical signs. The aim of this study was to investigate ORCS, CM/SM classification, and quantitative syrinx parameters in relation to progression of time (age) within individual Pomeranians.

**Materials and methods:**

Pomeranians with or without ORCS and with or without diagnoses of CM/SM were included that had undergone two (or more) MRI studies of the craniocervicothoracic region between January 2020 and June 2023. Classification of CM/SM and quantitative syrinx measurements were performed. Absolute values as well as ratios for syrinx height, width, and cross-sectional area were included for analysis.

**Results:**

A total of 19 Pomeranians were included in the study, of which 11 were male (58%) and 8 were female (42%). The median age at the time of MRI1 was 26 months (range 7–44 months). The median scan interval was 26 months (range 11–49 months). Eleven dogs (58%) were presented with ORCS at the time of MRI1, whereas the other 8 dogs (42%) had no ORCS at that time. At the time of MRI2, there were 17/19 dogs (89%) with ORCS and 2/19 dogs without ORCS (11%). Dogs were significantly more likely to have ORCS at MRI2 than MRI1 (*p* = 0. 0411). There was no significant difference between CM/SM classification at the time of MRI1 and MRI2. Significant differences were found between MRI1 and MRI2 for syrinx height (based on transverse images) (absolute value and ratio *P* = 0.0059), syrinx width (absolute value *P* = 0.1055, ratio *P* = 0.0039), and syrinx cross sectional area (absolute value *P* = 0.0195, ratio *P* = 0.0217).

**Discussion:**

There are differences in the presence or absence of ORCS as well as quantitative syrinx measurements in Pomeranians at different ages. This finding supports that longitudinal changes occur in the SM status of Pomeranians.

## Introduction

Chiari-like malformation (CM) and syringomyelia (SM) are disorders that, in dogs, affect mainly small and toy breeds. These disorders are linked to a great number of clinical and owner-reported clinical signs (ORCS) suggestive of pain (1–10). Due to the severity and/or persistent nature of such signs, owners may ultimately decide to euthanize their dog (8, 11, 12).

Increased age was associated with an increased risk of having SM in CKCS in several studies (10, 13–18). However, only few studies have shown that increased age was associated with syrinx size measured by different parameters such as width or total syrinx size (13, 14, 19). Studies that document an increased syrinx size related to increased age longitudinally (i.e., dogs were scanned twice at different ages) are scarce (14, 19). These studies documented progression of both SM and clinical signs in CKCS dogs.

An effect of age on the severity of SM as assessed by quantitative syrinx parameters can be of importance for several reasons. MRI scans are often performed for breeding purposes (“screenings”). Selecting dogs that are not affected by CM/SM could lead to a reduction in the prevalence of these disorders (15, 20). Regarding SM and the effect of age, if dogs are scanned at an early age, they could be misclassified as non-affected (healthy) or, when affected, graded differently at an early age than they would be at an older age. Another reason lies in assessing the effectiveness of treatment. There is, as of yet, no clear evidence for an effect of medical treatment on preventing syrinx development, halting progression, or decreasing syrinx size. An effect of age could influence or confound the results of studies on this matter. Studies on the longitudinal development of SM in the CKCS have already provided important insights into this subject for that particular breed (14, 19).

In our recently published study, we documented phenotypic characteristics of Pomeranians with and without CM/SM, including ORCS (10). We found an association between age and CM/SM classification. SM abnormal (affected) Pomeranians were significantly older than SM normal (non-affected) dogs. However, no associations were found between any of the included quantitative syrinx measurements and age. As that study did not longitudinally assess quantitative syrinx parameters within individuals over time, an effect of age on quantitative syrinx parameters could not be excluded.

The aim of this study was to investigate ORCS, CM/SM classification, and quantitative syrinx parameters in relation to progression of time (age) within individual Pomeranians. The hypotheses were that there would be:

1. A statistically significant differences in quantitative syrinx measurements at different ages.
2. A statistically significant difference in presence or absence of ORCS at different ages.
3. A statistically significant difference in CM/SM classification at different ages.

## Materials and methods

Case records of Pomeranians >6 months of age with or without ORCS and with or without diagnoses of CM/SM presented for MRI studies as well as clinical database software of two institutions [IVC Evidensia Referral Hospital Arnhem (Arnhem, The Netherlands), IVC Evidensia Referral Hospital Hart van Brabant (Waalwijk, The Netherlands)] were retrospectively reviewed for cases that had undergone two (or more) MRI studies of the craniocervicothoracic region between January 2020 and June 2023. Historical data and signalment [including country of origin, pedigree, sex, and age at time of first and second MRI scans (MRI1 and MRI2, respectively)] as well as information on treatment were collected from medical records, with those records including at least one consultation with a resident or diplomate in veterinary neurology. ORCS were recorded as present or absent at the time of MRI1 and MRI2. Specific ORCS were recorded as well, according to a standardized form and via questioning of the owners during consultation at the time of MRI1 and MRI2. This information was used to analyze for changes over time in ORCS. Time between MRI scans (scan interval MRI1-MRI2) and treatments were noted. MRI scans were randomly reviewed, and classifications of CM/SM and quantitative measurements were performed without knowledge of the scan being the first or second scan by one of the authors (KS).

MRI studies had been performed under general anesthesia (individualized anesthetic protocols). Dogs were positioned in sternal recumbency on the horizontal surface of the table with the head in a flexible or fixed coil, both resulting in elevation of the head of about 2–3 cm to the table. All MRI2 studies were performed with a high-field MRI scanner (1.5 T MRI, Canon Vantage Elan). MRI1 studies could have been acquired at a different institution with a different field strength including low-field MRI scanners. Dogs with only low-field MRI studies for MRI1 and MRI2 were excluded from this study. Sequences obtained included a minimum of sagittal T2W, sagittal T1W and transverse T2W or T1W sequences of the craniocervicothoracic region. At the involved institutions transverse images acquired were adjusted to center the syrinx, if visible. In dogs without a visible syrinx on sagittal images, transverse images were acquired at the level of the C2-C3 vertebrae. Dogs diagnosed with intracranial space-occupying lesions or space-occupying lesions in the vertebral canal were excluded. MRI studies with artifacts that did not allow for accurate measurements were excluded. Measurements were performed by the use of imaging software (Radiant DICOM viewer).

Classification of CM/SM and quantitative syrinx measurements were performed as previously described (10): CM0—no cerebellar herniation or impaction (cerebellar uvula rostral to foramen magnum); CM1—cerebellar impaction (cerebellar uvula on the line of the foramen magnum, no CSF present dorsal to the cervicomedullary junction) and non-rounded shape (e.g., flattened, pointed or indented by supraoccipital bone); CM2—cerebellar herniation (cerebellar uvula caudal to the line of the foramen magnum, no CSF present dorsal to the cervicomedullary junction); SM0—no SM; SM1—syrinx present and symmetric (i.e., circular, round syrinx); SM2—syrinx present and asymmetric (e.g., syrinx extending into a dorsal horn).

Quantitative measurements included:

- Maximum transverse syrinx width/spinal cord width ratio (STWR—transverse images).
- Maximum syrinx height/spinal cord height ratio on transverse images (SHRt—transverse images).
- Maximum syrinx cross-sectional area/spinal cord cross-sectional area ratio (SCSAR—transverse images).
- Maximum syrinx height/spinal cord height ratio on sagittal images (SHRs—sagittal images).

These measurements were based on T1-weighted (T1W) images. In addition to the ratios, absolute values for syrinx height [on transverse images (SHt) and sagittal images (SHs)], width [on transverse images (SWt)], and cross-sectional area [on transverse images (SCSA)] were included for analysis. Syrinx localization was noted as well: cervical, thoracic, extensive (both cervical and thoracic, continuous), or multifocal (both cervical and thoracic, discontinuous).

### Statistical analysis

Descriptive statistics are reported. A Shapiro-Wilk test was used to assess if the data followed a normal distribution. For continuous data, a paired Wilcoxon signed rank test was used to evaluate for differences between MRI1 and MRI2 and for categorical data a McNemar test was used to evaluate for differences between MRI1 and MRI2. *P*-values of < 0.05 were regarded as significant. Analyses were performed using Microsoft Excel and R v4.3.1.

## Results

Table 1 includes all patient data. A total of 19 Pomeranians were available for analysis, of which 11 were male (58%) and eight were female (42%). The median age at the time of MRI1 was 26 months (range 7–44 months). The median age at the time of MRI2 was 58 months (range 29–80 months). One dog was scanned 3 times at the ages of 21, 41, and 68 months, respectively. Of this dog, the first MRI scan was included as MRI1 and the third MRI scan was included as MRI2 for statistical analysis. The median scan interval was 26 months (range 11–49 months). Fifteen dogs (79%) were pedigree dogs, the other 4 (21%) were not (i.e., proper documentation was lacking or the dog was not registered at any of the national Kennel Clubs). Dogs originated from five different countries, including Russia 8 (42%), The Netherlands 6 (32%), Belgium 1 (5%), Italy 1 (5%), whilst of three dogs (16%), the country of origin was unknown. In five dogs (26%), MRI1 was performed at a different clinic with a low-field MRI scanner.

**Table 1:** Data of included Pomeranians.

**Case information**					**MRI1**					**MRI2**			
**Number**	**Sex**	**CoO**	**Ped**.	**Age at MRI1 in months**	**ORCS**	**CM**	**SM**	**SL**	**Scan interval in months**	**ORCS**	**CM**	**SM**	**SL**
1^*^	M	NL	Y	21	N	1	0		47	Y	1	1	Mu
2	F	NL	N	40	Y	0	1	Mu	27	Y	1	1	Mu
3	F	RU	Y	22	N	0	0		39	Y	1	0	
4	M	RU	Y	28	Y	1	2	Mu	24	Y	1	2	E
5	F	NL	Y	44	Y	1	0		19	Y	1	1	C
6	M	RU	Y	18	Y	1	1	Mu	12	Y	1	1	Mu
7	M	BU	Y	31	N	1	0		49	Y	1	0	
8	F	NL	Y	25	Y	1	0		31	Y	1	0	
9	F	RU	Y	28	N	1	0		14	Y	1	0	
10	M	RU	Y	17	Y	1	1	Mu	18	Y	1	1	Mu
11^*^	F	RU	Y	21	N	1	0		37	Y	1	1	Mu
12	F	Unknown	N	42	Y	0	0		17	Y	0	0	
13^*^	M	RU	Y	44	N	1	1	Mu	34	Y	1	1	Mu
14^*^	M	Unknown	Y	32	N	1	0		28	Y	1	0	
15	M	BE	N	7	Y	0	0		32	Y	0	0	
16	M	NL	N	33	Y	1	0		26	Y	1	0	
17	F	NL	Y	11	N	1	1	Mu	18	Y	1	2	Mu
18^*^	M	IT	Y	23	N	1	2	C	11	N	1	2	C
19	M	RU	Y	26	Y	1	0		16	N	1	0	

BE, Belgium; BU, Bulgaria; C, cervical SM; CM, Chiari-like malformation; E, extensive SM; F, female; IT, Italy; M, male; MRI1, first MRI study; MRI2, second MRI study; Mu, multifocal SM; N, no; NL, The Netherlands; ORCS, owner-reported clinical signs; Ped, pedigree; RU, Russia; SL, syrinx localization; SM, syringomyelia; Y, yes.

^*^MRI1 was a low-field MRI study.

**Figure 1:**
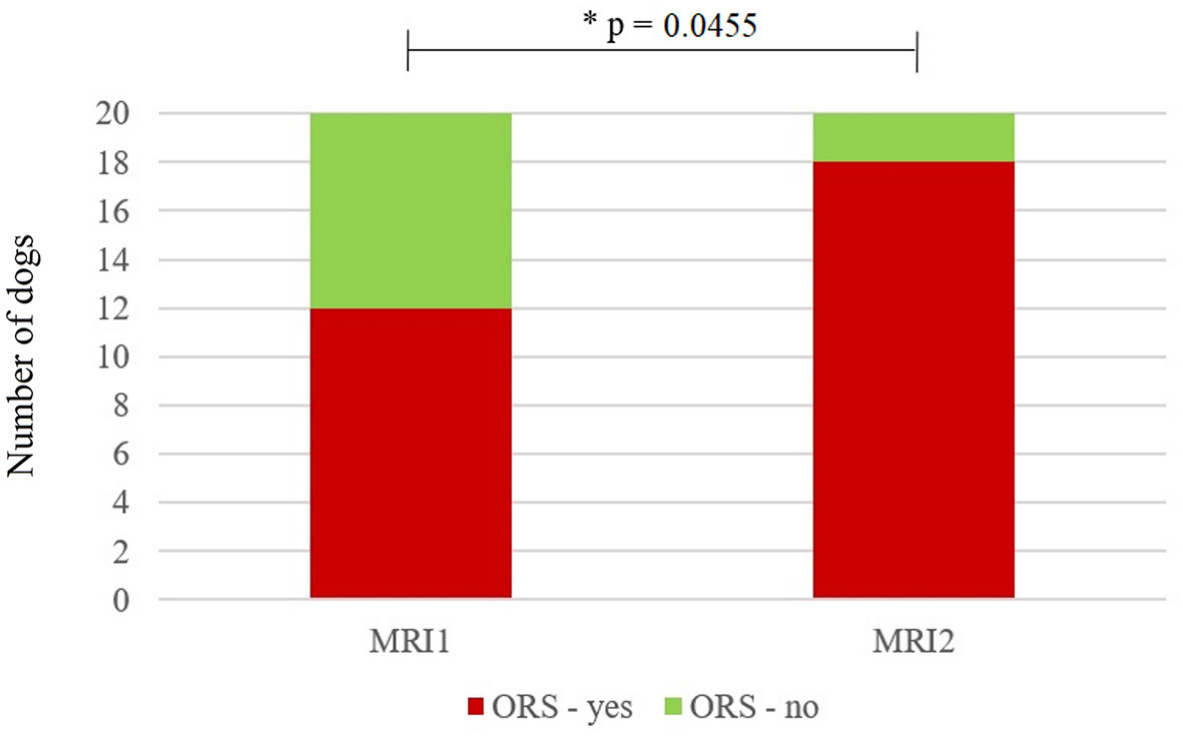
Stacked bar graph showing the number of dogs with owner-reported clinical signs (ORCS—yes) and without ORCS (ORCS—no) at the time of the first magnetic resonance imaging scan (MRI1) and the second MRI scan (MRI2). Significantly more dogs had ORCS at MRI2 (*p* = 0.0411). ^*^Statistically significant (*p* < 0.05).

Eleven dogs (58%) were presented with ORCS at the time of MRI1, whereas the other eight dogs (42%) had no ORCS at that time. At the time of MRI2, seven dogs previously not having ORCS were now presented with ORCS and 1 dog previously having ORCS did not have ORCS anymore, giving a total number of 17/19 dogs (89%) with ORCS and 2/19 dogs without ORCS (11%). Dogs were significantly more likely to have ORCS at MRI2 than MRI1 (*p* = 0.0455) (Figure 1). The main reason for performing MRI2 was a change in ORCS (17/19, 89%), while no specific reason was identified in patient records for two dogs (11%).

Details on specific ORCS, those present at the time of MRI and those present or persisting at the time of MRI2 are included in Tables 2A, B. Six out of 11 dogs (55%) that had ORCS at MRI1 had developed previously unreported, additional ORCS at MRI2, whilst 4/11 dogs (36%) that had ORCS at MRI1 had retained the same ORCS at MRI2, and 1 dog (9%) that had ORCS at MRI1 did not have ORCS anymore at MRI2.

**Table 2A:** Owner-reported clinical signs at the time of first MRI (MRI1) and second MRI (MRI2) of dogs 1–10.

**Case**	**1**		**2**		**3**		**4**		**5**		**6**		**7**		**8**		**9**		**10**	
**MRI**	**1**	**2**	**1**	**2**	**1**	**2**	**1**	**2**	**1**	**2**	**1**	**2**	**1**	**2**	**1**	**2**	**1**	**2**	**1**	**2**
**ORCS** ▾																				
Air licking			X	X			X	X	X	X					X	X			X	X
Fly catching or tail chasing																				
Head shaking		X	X	X		X										X		X		
Hyperexcitability										X										
Lethargy							X	X	X	X				X						
Licking front and/or hind limbs		X				X										X		X	X	X
Phantom scratching							X	X	X	X		X							X	X
Provoked signs of pain														X		X		X		
Scratching with skin contact, rubbing head or ears, or both		X					X	X	X	X	X	X		X	X	X			X	X
Spontaneous signs of pain		X	X	X						X		X		X						
Vocalization												X								
Weakness				X																

**Table 2B:** Owner-reported clinical signs at the time of first MRI (MRI1) and second MRI (MRI2) of dogs 11–20.

**Case**	**11**		**12**		**13**		**14**		**15**		**16**		**17**		**18**		**19**	
**MRI**	**1**	**2**	**1**	**2**	**1**	**2**	**1**	**2**	**1**	**2**	**1**	**2**	**1**	**2**	**1**	**2**	**1**	**2**
**ORCS** ▾																		
Air licking		X			X	X		X										
Fly catching or tail chasing				X		X		X										
Head shaking		X												X			X	
Hyperexcitability																		
Lethargy																	X	
Licking front and/or hind limbs		X		X														
Phantom scratching																		
Provoked signs of pain			X			X												
Scratching with skin contact, rubbing head or ears, or both		X	X	X				X			X	X		X			X	
Spontaneous signs of pain		X	X			X			X	X							X	
Vocalization						X												
Weakness																		

Information on treatment per case is included in Supplementary Table 1. Medication and supplements used included amitriptyline (9/19), furosemide (6/19), cannabidiol (3/19), meloxicam (2/19), carprofen (2/19), tramadol (2/19), gabapentin (2/19), and firocoxib (1/19).

### CM/SM classification

Table 1 includes CM/SM classifications at the time of MRI1 and MRI2 for all patients. CM classification was different between MRI1 and MRI2 for 2 dogs, being classified as CM0 at MRI1 and CM1 at MRI2. There was no significant difference between CM classification at the time of MRI1 and MRI2 (*p* = 0.4795). SM classification was different between MRI1 and MRI2 for four dogs, three dogs being classified as SM0 at MRI1 and SM1 at MRI2, and one dog being classified as SM1 at MRI1 and SM2 at MRI2. There was no significant difference between SM classification at the time of MRI1 and MRI2 (*p* = 0.1336). Localization of SM was different between MRI1 and MRI2 for four dogs. Two dogs with no SM at MRI1 had multifocal SM as MRI2, one dog had no SM at MRI1 and cervical SM at MRI2, and 1 dog had multifocal SM at MRI1 and extensive SM at MRI2.

**Figure 2:**
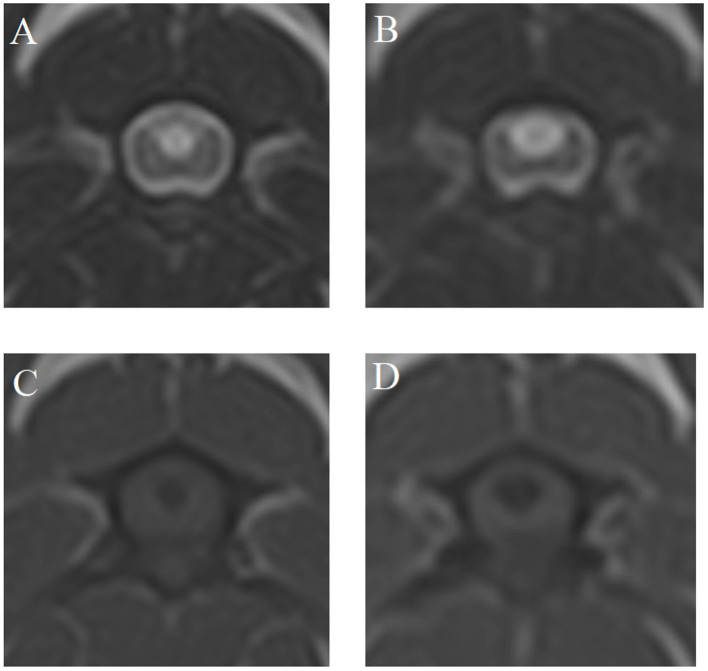
T2- **(A, B)** and T1-weighted **(C, D)** magnetic resonance images (MRI) at the time of MRI1 **(A, C)** and MRI2 **(B, D)** of case 4—scan interval: 24 months.

Figure 2 shows an example of MRI findings at MRI1 and MRI2 of one dog with a scan interval of 24 months.

### Quantitative syrinx parameters

**Figure 3:**
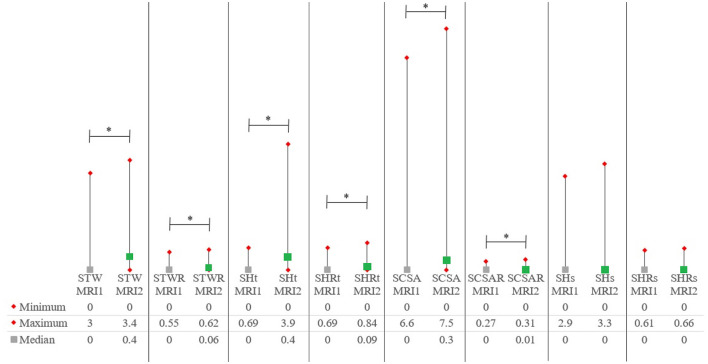
Medians and ranges for quantitative syrinx measurements. *P*-values are included in Tables 2A, B. MRI1, first magnetic resonance imaging study; MRI2, second MRI study; SCSA, maximum syrinx cross-sectional area; SCSAR, maximum syrinx cross-sectional area/spinal cord cross-sectional area ratio; SHt, maximum syrinx height measured on transverse images; SHRt, maximum syrinx height/spinal cord height ratio measured on transverse images; STW, maximum transverse syrinx width; STWR, maximum transverse syrinx width/spinal cord width ratio; SHRs, maximum syrinx height measured on sagittal images; SHRs, maximum syrinx height/spinal cord height ratio measured on sagittal images. ^*^Statistically significant (*p* < 0.05).

Supplementary Table 2 includes all quantitative syrinx measurements at the time of MRI1 and MRI2 for all patients. Quantitative syrinx measurements at MRI1 and MRI2 are included in Table 3. Significant differences were found between MRI1 and MRI2 for all parameters except SHs and SHRs. Except for SHs and SHRs, all measurements were significantly higher at MRI2 compared to MRI1 (Figure 3).

**Table 3:** Descriptive statistics and *P*-values (significant marked with ^*^) of Wilcoxon rank sum tests for all quantitative syrinx measurements.

**Parameter**	**MRI1**	**MRI2**	***P*-value**
STW (mm)	0.0 (0.0–3.0)	0.4 (0.0–3.4)	0.0059^*^
STWR	0.00 (0.00–0.55)	0.06 (0.00–0.62)	0.0059^*^
SHt (mm)	0.0 (0.0–2.9)	0.4 (0.0–3.9)	0.0020^*^
SHRt	0.00 (0.00–0.69)	0.09 (0.00–0.84)	0.0039^*^
SCSA (mm^2^)	0.0 (0.0–6.6)	0.3 (0.0–7.5)	0.0195^*^
SCSAR	0.00 (0.00–0.27)	0.01 (0.00–0.31)	0.0217^*^
SHs (mm)	0.0 (0.0–2.9)	0.0 (0.0–3.3)	0.2131
SHRs	0.00 (0.00–0.61)	0.00 (0.00–0.66)	0.1537

Median and range are provided for all parameters. SCSA, maximum syrinx cross-sectional area; SCSAR, maximum syrinx cross-sectional area/spinal cord cross-sectional area ratio; SHt, maximum syrinx height measured on transverse images; SHRt, maximum syrinx height/spinal cord height ratio measured on transverse images; STW, maximum transverse syrinx width; STWR, maximum transverse syrinx width/spinal cord width ratio; SHRs, maximum syrinx height measured on sagittal images; SHRs, maximum syrinx height/spinal cord height ratio measured on sagittal images.

## Discussion

In this longitudinal study, syrinx size as based on multiple quantitative parameters increased over time within individual Pomeranian dogs. Therefore, our first hypothesis is accepted. This finding is consistent with previous longitudinal studies in the CKCS that showed increased size parameters between MRI1 and MRI2 (14, 19).

As in our previous study (10), we included several types of quantitative assessments in our study. T1W and T2W measurements are highly correlated, but T2W-based measurements may be hampered by difficulty in differentiating between spinal cord oedema and true syrinx margins (10, 21). Therefore, we elected to include only T1W-based measurements in this study. SM measurements are often recorded absolutely (e.g., a syrinx diameter of 2.2 mm), or dogs are classified in groups with specific measurement categories (e.g., < 1.0 mm, 1.0–1.9 mm, et cetera), or dogs are classified as having SM or not based on size (e.g., >2 mm) (6, 7, 14, 19, 22–29). Few studies provided relative syrinx sizes (e.g., syrinx height: C3 vertebral depth or the spinal cord itself) (9, 13, 19). Acknowledging that body size varies between individual dogs, absolute measurements would be less useful than ratios. Using a particular vertebra as a reference also has limitations. As the diameter of the spinal cord varies between different locations (e.g., intumescence vs. non-intumescence), this will result in a lower ratio when a syrinx is measured in a non-intumescence compared to an intumescence segment of the spinal cord. For these reasons, the authors prefer to report ratios of syrinx measurements compared to the spinal cord itself (for height, width, cross-sectional area) as a reference (10, 13). However, when a longitudinal study is performed, absolute measurement of syrinx diameter may still be useful, as an absolute change in syrinx size at different time points for the same individual are relevant. This is reflected in our results by the fact that the corresponding absolute measurements of all ratios that showed significant differences between MRI1 and MRI2 were significantly different between MRI1 and MRI2 as well.

While most measurements, including SHt, SHRt, SWt, SWRt, SCSA, and SCSAR were significantly different between MRI1 and MRI2, SHs and SHRs were not. Measuring syrinx height on sagittal MR images can be inaccurate since acquiring exactly midsagittal images can be troublesome and not guaranteed. Thus, syrinx height may be underestimated when it is not sagittally represented at its full extent. Since all other measurements, particularly SHt and SHRt, were significantly different between MRI1 and MRI2, transverse measurements are recommended to measure syrinx height.

Dogs were significantly more likely to be presented with ORCS at MRI2 (89%) than MRI1 (58%). Hence, our second hypothesis is accepted. In a previous study, a common reason for CKCS being presented for a second MRI study was “poor control of original complaint” (14). In another study on the CKCS (19), of the 16 dogs that were originally asymptomatic, 56% showed worsening and 31% had unchanged signs at repeat evaluations, while only 13% had improved. Thirty-two percent of 38 initially asymptomatic dogs were symptomatic at the time of repeat evaluation. Both those studies in the CKCS also found increased syrinx sizes between MRI1 and MRI2 (14, 19). In line with these findings, our results indicate that Pomeranians are more likely to be presented with ORCS linked to CM/SM at later stages in life. Having no ORCS at an early age would not preclude the possibility of having ORCS at a later age.

There are several considerations that deserve to be mentioned here. The possible effect of the various medications that were administered to the dogs in these other longitudinal studies is not accounted for (14, 19). In our study, treatment varied between cases and was chosen at the discretion of the involved clinician and/or owner. Currently, there are no studies evaluating the effect or effectiveness of the various medications given to affected dogs in relationship to CM/SM quantitative parameters over time. Of the medications used in the dogs included in this study, furosemide is of particular interest as this diuretic may influence development of a syrinx and syrinx size, whereas the other types of medications most likely do not. The limited number of dogs administered furosemide in this study precluded statistical evaluation of the effect of furosemide on syrinx size. SM is referred to as a progressive disorder (1, 2, 6, 7, 12, 14, 19), a statement that is underlined by our results. However, based on the collective clinical experience of the authors, a decrease in syrinx size over time is possible as well. Therefore, instead of referring to SM as a “progressive disorder”, we prefer the term “dynamic” when describing the nature of SM in dogs. Referring to SM as “progressive” would exclude the possibility of improvement or decrease in syrinx size over time. It may be better to say “typically progressive”, based on the results of our study and previous studies (14, 19). To the authors' opinion, this is an important consideration when assessing development of SM in dogs. Whether changes in syrinx dimensions are affected by medications often given to dogs with SM is unclear at this time. Future studies may be able to further asses the effect of this medication on syrinx size.

There were no significant differences in CM/SM classifications in this study. Consequently, our third hypothesis was rejected. Nevertheless, our results do show that both may change over time within individuals. As CM/SM classification was different at different ages in a previous study with a much larger sample size of 796 dogs (10), this likely reflects a type II error in the current smaller sample size study or may be due to differences in methods. We must also take into consideration that there are several limitations to longitudinal studies such as this and previous longitudinal studies in the CKCS (14, 19). As the age at MRI1 varied between dogs, the state of CM/SM is analyzed at different stages of its development. Moreover, scan interval varied between dogs as well. If a dog is scanned at relatively old age (e.g., 5 years), changes in syrinx parameters at an even later time may be much less pronounced than when they would have been scanned the first time at a young age (e.g., 1 year). Although syrinx size increased with age in this study, such an increase in size is not limitless. A ratio reflects this inherently, as a ratio of 1.00 (100% syrinx, no spinal cord—an unrealistic scenario of course) is the maximum ratio. Likewise, absolute syrinx measurements will be capped at some point. Acknowledging such limitations, the changing nature of SM is something that has to be dealt with in studies focusing on objective assessments thereof.

As in former studies, some forms of selection bias are represented in this study that can influence the results (14, 19). Since this was a retrospective study and owners voluntarily presented their dogs for a second MRI study, there was a likelihood that dogs with progressive signs would be presented for a second study instead of dogs that were doing well. On the other hand, dogs that had shown progressive signs or had such severe signs that owners elected euthanasia at an earlier time point would be less likely to have been presented for a second MRI scan. Future prospective studies could address such issues by including all dogs for a second MRI scan at a set point in time. Several considerations, e.g., financial, ethical, and logistical, would need to be addressed in the study designs.

A limitation of this study compared to previous longitudinal studies is the inclusion of some low-field MRI studies for MRI1 in a minority of patients. Rather than excluding these patients, we included them as studies have shown that low-field MRI can be used to detect CM/SM (30). Of the 5 dogs that had a low-field MRI1, all had a high-field MRI2 and only 2/5 dogs were classified differently at MRI2, being SM0 and SM1 at MRI1 and MRI2, respectively. When low-field MRI studies are evaluated for CM/SM, T1W images are anecdotally more useful and have been used in previous studies (6, 31). There are no specific studies evaluation low- vs. high-field MRI studies, but high-field MRI studies are the gold standard for diagnosing CM/SM in dogs (1, 31). Intermodality (low- vs. high-field) agreement studies of quantitative SM measurements could provide more information on the reliability of performing these measurements and comparing them. Lacking those studies, we cannot ascertain the effect this had on comparing results of measurements between these modalities for five of the included dogs in this study. Another limitation is the small sample size in this study, which applies to the previous studies in the CKCS as well. Future studies including a prospective design, larger sample size, and a standardized scan interval would be beneficial to evaluate longitudinal development of these disorders in dogs. Finally, when performing scans at different time points of the same individual dogs, even though the positioning was performed in the same general way, differences in exact positioning cannot be excluded. Also, the exact planning parameters of the acquired MRI sequences (e.g., slopes of transverse images) can be subject to change between the different scans. These factors are likely to influence classification as well as quantitative measurement results.

In conclusion, this longitudinal study found differences in the presence or absence of ORCS as well as quantitative syrinx measurements in Pomeranians at different ages. These results underline the merits of performing scans at different ages within individual dogs. Also, this finding supports that SM in the Pomeranian, like in the CKCS, typically is a progressive disorder, with variable clinical presentation and imaging findings at different ages within individual dogs. This should be taken into account when dogs are presented for CM/SM related ORCS as well as in screening protocols and breeding selection recommendations.

## Data availability statement

The original contributions presented in the study are included in the article/Supplementary material, further inquiries can be directed to the corresponding authors.

## Ethics statement

The animal studies were approved by the Animal Welfare Body Utrecht, Utrecht University, The Netherlands. The studies were conducted in accordance with the local legislation and institutional requirements. Written informed consent was obtained from the owners for the participation of their animals in this study.

## Author contributions

KS: Conceptualization, Data curation, Formal analysis, Funding acquisition, Investigation, Methodology, Project administration, Resources, Visualization, Writing – original draft, Writing – review & editing. IC: Conceptualization, Supervision, Validation, Writing – review & editing. PM: Conceptualization, Data curation, Funding acquisition, Investigation, Methodology, Project administration, Resources, Supervision, Writing – review & editing.

## References

[1] Hechler AC Moore SA. Understanding and treating chiari-like malformation and syringomyelia in dogs. Top Companion Anim Med. (2018) 33:1–11. 10.1053/j.tcam.2018.03.002 29793722

[2] Knowler SP McFadyen AK Freeman C Kent M Platt SR Kibar Z. Quantitative analysis of Chiari-like malformation and syringomyelia in the Griffon Bruxellois dog. PLoS ONE. (2014) 9:e88120. 10.1371/journal.pone.0088120 24533070 PMC3922758

[3] Loughin CA. Chiari-like malformation. Vet Clin North Am Small Anim Pract. (2016) 46:231–42. 10.1016/j.cvsm.2015.10.002 26631589

[4] Rusbridge C Knowler SP. The need for head space: brachycephaly and cerebrospinal fluid disorders. Life. (2021) 11:139. 10.3390/life11020139 33673129 PMC7918167

[5] Rusbridge C Knowler SP Pieterse L McFadyen AK. Chiari-like malformation in the Griffon Bruxellois. J Small Anim Pract. (2009) 50:386–93. 10.1111/j.1748-5827.2009.00744.x 19689665

[6] Rusbridge C McFadyen AK Knower SP. Behavioral and clinical signs of Chiari-like malformation-associated pain and syringomyelia in Cavalier King Charles spaniels. J Vet Intern Med. (2019) 33:2138–50. 10.1111/jvim.15552 31290195 PMC6766577

[7] Nalborczyk ZR McFadyen AK Jovanovik J Tauro A Driver CJ Fitzpatrick N. characteristics for “phantom” scratching in canine syringomyelia. BMC Vet Res. (2017) 13:340. 10.1186/s12917-017-1258-2 29145838 PMC5691609

[8] Sanchis-Mora S Pelligand L Thomas CL Volk HA Abeyesinghe SM Brodbelt DC. Dogs attending primary-care practice in England with clinical signs suggestive of Chiari-like malformation/syringomyelia. Vet Rec. (2016) 179:436. 10.1136/vr.103651 27534983

[9] Sparks CR Cerda-Gonzalez S Griffith EH Lascelles BDX Olby NJ. Questionnaire-based analysis of owner-reported scratching and pain signs in Cavalier King Charles spaniels screened for Chiari-like malformation and Syringomyelia. J Vet Intern Med. (2018) 32:331–9. 10.1111/jvim.14856 29105875 PMC5787193

[10] Santifort KM Carrera I Bossens K Mandigers PJJ. Phenotypic characterization of Pomeranians with or without Chiari-like malformation and syringomyelia. Front Vet Sci. (2023) 10:1320942. 10.3389/fvets.2023.1320942 38169622 PMC10758411

[11] Yu Y Wilson B Masters S van Rooy D McGreevy PD. Mortality resulting from undesirable behaviours in dogs aged three years and under attending primary-care veterinary practices in Australia. Animals. (2021) 11:493. 10.3390/ani11020493 33668532 PMC7918417

[12] Plessas IN Rusbridge C Driver CJ Chandler KE Craig A McGonnell IM. Long-term outcome of Cavalier King Charles spaniel dogs with clinical signs associated with Chiari-like malformation and syringomyelia. Vet Rec. (2012) 171:501. 10.1136/vr.100449 23100307

[13] Loderstedt S Benigni L Chandler K Cardwell JM Rusbridge C Lamb CR. Distribution of syringomyelia along the entire spinal cord in clinically affected Cavalier King Charles Spaniels. Vet J. (2011) 190:359–63. 10.1016/j.tvjl.2010.12.002 21216639

[14] Driver CJ De Risio L Hamilton S Rusbridge C Dennis R McGonnell IM. Changes over time in craniocerebral morphology and syringomyelia in cavalier King Charles spaniels with Chiari-like malformation. BMC Vet Res. (2012) 8:215. 10.1186/1746-6148-8-215 23136935 PMC3514376

[15] Wijnrocx K Van Bruggen LWL Eggelmeijer W Noorman E Jacques A Buys N. Twelve years of chiari-like malformation and syringomyelia scanning in Cavalier King Charles Spaniels in the Netherlands: Towards a more precise phenotype. PLoS ONE. (2017) 12:e0184893. 10.1371/journal.pone.0184893 28934242 PMC5608246

[16] Couturier J Rault D Cauzinille L. Chiari-like malformation and syringomyelia in normal cavalier King Charles spaniels: a multiple diagnostic imaging approach. J Small Anim Pract. (2008) 49:438–43. 10.1111/j.1748-5827.2008.00578.x 18631225

[17] Knowler SP McFadyen AK Rusbridge C. Effectiveness of breeding guidelines for reducing the prevalence of syringomyelia. Vet Rec. (2011) 169:681. 10.1136/vr.100062 21998144

[18] Parker JE Knowler SP Rusbridge C Noorman E Jeffery ND. Prevalence of asymptomatic syringomyelia in Cavalier King Charles spaniels. Vet Rec. (2011) 168:667. 10.1136/vr.d1726 21672954

[19] Cerda-Gonzalez S Olby NJ Griffith EH. Longitudinal study of the relationship among craniocervical morphology, clinical progression, and syringomyelia in a cohort of Cavalier King Charles Spaniels. J Vet Intern Med. (2016) 30:1090–8. 10.1111/jvim.14362 27311874 PMC5094541

[20] Limpens C Smits VTM Fieten H Mandigers PJJ. The effect of MRI-based screening and selection on the prevalence of syringomyelia in the Dutch and Danish Cavalier King Charles Spaniels. Front Vet Sci. (2024) 11:1326621. 10.3389/fvets.2024.1326621 38348108 PMC10859423

[21] Akiyama Y Koyanagi I Yoshifuji K Murakami T Baba T Minamida Y. Interstitial spinal-cord oedema in syringomyelia associated with Chiari type 1 malformations. J Neurol Neurosurg Psychiatry. (2008) 79:1153–8. 10.1136/jnnp.2007.133959 18403441

[22] Schulze S Refai M Deutschland M Failing K Schmidt M. Prevalence of syringomyelia in clinically unaffected Cavalier King Charles Spaniels in Germany (2006-2016). Tierarztl Prax Ausg K Kleintiere Heimtiere. (2018) 46:157–62. English. 10.15654/TPK-170725 29898477

[23] Knowler SP Dumas E Spiteri M McFadyen AK Stringer F Wells K. Facial changes related to brachycephaly in Cavalier King Charles Spaniels with Chiari-like malformation associated pain and secondary syringomyelia. J Vet Intern Med. (2020) 34:237–46. 10.1111/jvim.15632 31691386 PMC6979263

[24] Bva T. Chiari Malformation/Syringomyelia Scheme (CM/SM Scheme). (2023). Available online at: https://www.bva.co.uk/canine-health-schemes/cmsm-scheme/ (accessed August 17, 2023).

[25] Hechler AC Hostnik ET Cook LB Cole LK Moore SA. Mechanical quantitative sensory testing in cavalier King Charles spaniels with and without syringomyelia. BMC Vet Res. (2020) 16:94. 10.1186/s12917-020-02313-7 32197618 PMC7085174

[26] Sparks CR Gorney A Williams K Griffith EH Cerda-Gonzalez S Lascelles BDX. Investigation of sensory thresholds in Cavalier King Charles Spaniels with and without Chiari-like malformations and syringomyelia. J Vet Intern Med. (2018) 32:2021–8. 10.1111/jvim.15297 30307645 PMC6272044

[27] Thoefner MS Westrup U Toft N Bjerrum OJ Berendt M. Mechanical sensory threshold in Cavalier King Charles spaniels with syringomyelia-associated scratching and control dogs. Vet J. (2019) 246:92–7. 10.1016/j.tvjl.2019.01.011 30902196

[28] Rusbridge C Carruthers H Dubé MP Holmes M Jeffery ND. Syringomyelia in cavalier King Charles spaniels: the relationship between syrinx dimensions and pain. J Small Anim Pract. (2007) 48:432–6. 10.1111/j.1748-5827.2007.00344.x 17608656

[29] Kiviranta AM Rusbridge C Laitinen-Vapaavuori O Hielm-Björkman A Lappalainen AK Knowler SP. Syringomyelia and craniocervical junction abnormalities in Chihuahuas. J Vet Intern Med. (2017) 31:1771–81. 10.1111/jvim.14826 28892202 PMC5697179

[30] Kromhout K van Bree H Broeckx BJ Bhatti S De Decker S Polis I. Low-field magnetic resonance imaging and multislice computed tomography for the detection of cervical Syringomyelia in dogs. J Vet Intern Med. (2015) 29:1354–9. 10.1111/jvim.13579 26249824 PMC4858036

[31] Rusbridge C Stringer F Knowler SP. Clinical application of diagnostic imaging of chiari-like malformation and Syringomyelia. Front Vet Sci. (2018) 5:280. 10.3389/fvets.2018.00280 30547039 PMC6279941

