# Supplementary material for: Longitudinal assessment of syringomyelia in Pomeranians
Source: Front Vet Sci. 2024 May 1;11:1364464. doi: 10.3389/fvets.2024.1364464 (PMC11094614; doi:10.3389/fvets.2024.1364464)
Supplement: Supplementary file 1 [file Data_Sheet_1.PDF]

Supplementary Material

Longitudinal assessment of syringomyelia in Pomeranians

Koen M. Santifort[1,2,3*], Ines Carrera[4], Paul J.J. Mandigers[1,3]

[1]IVC Evidensia Referral Hospital Arnhem, Neurology, Arnhem, The Netherlands

[2]IVC Evidensia Referral Hospital Hart van Brabant, Neurology, Waalwijk, The Netherlands

[3]Expertise Centre of Genetics, Department of Clinical Sciences, Faculty of Veterinary Medicine, Utrecht University, Utrecht, The Netherlands

[4]Vet Oracle Teleradiology, Norfolk, United Kingdom

*** Correspondence:**
koen.santifort@evidensia.nl
Koen M. Santifort

Supplementary table 1: Treatment information per case.

Case number	Treatment pre-MRI1	Treatment post-MRI1	Treatment pre-MRI2	Treatment post-MRI2
1	-	-	-	Furosemide<br>Amitriptyline
2	-	Furosemide<br>Amitriptyline	Furosemide<br>Amitriptyline<br>Cannabidiol	Furosemide<br>Amitriptyline<br>Cannabidiol
3	-	-	-	-
4	-	Amitriptyline	Amitriptyline<br>Cannabidiol	Amitriptyline<br>Cannabidiol
5	-	-	Gabapentin<br>Meloxicam	Meloxicam<br>Amitriptyline<br>Furosemide
6	Meloxicam	Meloxicam<br>Amitriptyline	Amitriptyline<br>Furosemide	Amitriptyline<br>Furosemide
7	-	-	-	Amitriptyline
8	-	-	-	-
9	-	Carprofen	Carprofen<br>Tramadol	Carprofen<br>Tramadol
10	-	Amitriptyline	Amitriptyline<br>Cannabidiol	Amitriptyline<br>Cannabidiol
11	-	-	Amitriptyline	Amitriptyline<br>Tramadol
12	-	-	-	-
13	-	Furosemide	Furosemide	Furosemide<br>Amitriptyline<br>Gabapentin
14	-	-	-	-
15	-	-	-	-
16	-	-	-	-
17	-	-	Amitriptyline<br>Firocoxib	Amitriptyline
18	-	-	-	-
19	-	Furosemide<br>Carprofen	Furosemide<br>Carprofen	Furosemide<br>Carprofen

Supplementary table 2: Quantitative syrinx measurements for included dogs. SCSA = maximum syrinx cross-sectional area, SCSAR = maximum syrinx cross-sectional area/spinal cord cross-sectional area ratio, SHt = maximum syrinx height measured on transverse images, SHRt = maximum syrinx height/spinal cord height ratio measured on transverse images, STW = maximum transverse syrinx width, STWR = maximum transverse syrinx width/spinal cord width ratio, SHRs = maximum syrinx height measured on sagittal images, SHRs maximum syrinx height/spinal cord height ratio measured on sagittal images.

**Case**	**MRI1**							
	**STW (mm)**	**STWR**	**SHt (mm)**	**SHRt**	**SCSA (mm^2)**	**SCSAR**	**SHs (mm)**	**SHRs**
**1**	-	-	-	-	-	-	-	-
**2**	2.7	0.42	2.7	0.69	4.9	0.19	2.6	0.53
**3**	-	-	-	-	-	-	-	-
**4**	2.3	0.55	2.2	0.68	3.5	0.27	1.7	0.39
**5**	-	-	-	-	-	-	-	-
**6**	2.4	0.46	2.4	0.47	5.0	0.26	2.4	0.47
**7**	-	-	-	-	-	-	-	-
**8**	-	-	-	-	-	-	-	-
**9**	-	-	-	-	-	-	-	-
**10**	1.1	0.24	1.3	0.36	1.0	0.08	0.5	0.14
**11**	-	-	-	-	-	-	-	-
**12**	-	-	-	-	-	-	-	-
**13**	1.2	0.22	1.7	0.42	2.4	0.13	1.6	0.34
**14**	-	-	-	-	-	-	-	-
**15**	-	-	-	-	-	-	-	-
**16**	-	-	-	-	-	-	-	-
**17**	2.0	0.35	1.5	0.41	2.9	0.21	0.8	0.23
**18**	3.0	0.44	2.9	0.54	6.6	0.23	2.9	0.61
**19**	-	-	-	-	-	-	-	-

Supplementary table 3 continued

**Case**	**MRI2**							
	**STW (mm)**	**STWR**	**SHt (mm)**	**SHRt**	**SCSA (mm^2)**	**SCSAR**	**SHs (mm)**	**SHRs**
**1**	1.3	0.21	2.7	0.63	2.7	0.11	1.3	0.27
**2**	2.5	0.38	3.0	0.70	5.2	0.21	1.7	0.39
**3**	-	-	-	-	-	-	-	-
**4**	2.7	0.62	3.9	0.76	6.7	0.31	3.3	0.66
**5**	0.7	0.11	0.8	0.17	0.5	0.02	0.0	0.00
**6**	3.2	0.48	3.0	0.67	6.8	0.27	1.9	0.41
**7**	-	-	-	-	-	-	-	-
**8**	-	-	-	-	-	-	-	-
**9**	-	-	-	-	-	-	-	-
**10**	1.3	0.30	1.6	0.41	1.6	0.12	1.0	0.25
**11**	1.4	0.24	1.0	0.28	1.9	0.10	1.0	0.27
**12**	-	-	-	-	-	-	-	-
**13**	1.6	0.28	1.8	0.42	2.5	0.10	1.0	0.26
**14**	-	-	-	-	-	-	-	-
**15**	-	-	-	-	-	-	-	-
**16**	-	-	-	-	-	-	-	-
**17**	2.7	0.50	3.8	0.84	6.9	0.30	2.6	0.54
**18**	3.4	0.49	3.3	0.57	7.5	0.26	3.1	0.64
**19**	-	-	-	-	-	-	-	-

Definitions of ORCS

Air licking
Licking the air frequently and repetitively.

Fly catching or tail chasing
Repetitively, episodically biting the air (as if there is a fly to catch) or chasing the tail.

Head shaking
Unprovoked head shaking.

Hyperexcitability
E.g. overreaction to external stimuli, inability to relax, episodically running in circles.

Lethargy
E.g. increased sleepiness, decreased excitement.

Licking front and/or hind limbs
Excessively and/or repetitively licking front and/or hind limbs without identifiable skin disease.

Phantom scratching
Scratching towards the neck area, but not making skin-contact.

Provoked signs of pain
Any sign that suggested to the owner the dog may be or have been experiencing pain that was elicited by external stimuli or activities (e.g. by being picked up, touched, or when asked to perform certain activities (playing)).

Scratching with skin contact, rubbing head or ears, or both
Scratching head, neck or shoulder areas with front or hind limbs. Rubbing head or ears with front limbs, on walls, or on the floor.

Spontaneous signs of pain
Any sign that suggested to the owner the dog may be or have been experiencing pain that is not elicited by external stimuli or activities (e.g. touch).

Vocalization
Yelping, yelling, screaming, barking (without identifiable other reasons).

Weakness
Tripping, falling, difficulty walking or supporting weight.
